# Fuzzy Model for the Automatic Recognition of Human Dendritic Cells

**DOI:** 10.3390/jimaging9010013

**Published:** 2023-01-06

**Authors:** Marwa Braiki, Kamal Nasreddine, Abdesslam Benzinou, Nolwenn Hymery

**Affiliations:** 1 ENIB, UMR CNRS 6285 LabSTICC, 29238 Brest, France; 2 Univ Brest, Laboratoire Universitaire de Biodiversité et Écologie Microbienne, 29280 Plouzané, France

**Keywords:** classification, dendritic cells, morphology, SVM, FLD, fuzzy logic

## Abstract

**Background and objective:** Nowadays, foodborne illness is considered one of the most outgrowing diseases in the world, and studies show that its rate increases sharply each year. Foodborne illness is considered a public health problem which is caused by numerous factors, such as food intoxications, allergies, intolerances, etc. Mycotoxin is one of the food contaminants which is caused by various species of molds (or fungi), which, in turn, causes intoxications that can be chronic or acute. Thus, even low concentrations of Mycotoxin have a severely harmful impact on human health. It is, therefore, necessary to develop an assessment tool for evaluating their impact on the immune response. Recently, researchers have approved a new method of investigation using human dendritic cells, yet the analysis of the geometric properties of these cells is still visual. Moreover, this type of analysis is subjective, time-consuming, and difficult to perform manually. In this paper, we address the automation of this evaluation using image-processing techniques. **Methods:** Automatic classification approaches of microscopic dendritic cell images are developed to provide a fast and objective evaluation. The first proposed classifier is based on support vector machines (SVM) and Fisher’s linear discriminant analysis (FLD) method. The FLD–SVM classifier does not provide satisfactory results due to the significant confusion between the inhibited cells on one hand, and the other two cell types (mature and immature) on the other hand. Then, another strategy was suggested to enhance dendritic cell recognition results that are emitted from microscopic images. This strategy is mainly based on fuzzy logic which allows us to consider the uncertainties and inaccuracies of the given data. **Results:** These proposed methods are tested on a real dataset consisting of 421 images of microscopic dendritic cells, where the fuzzy classification scheme efficiently improved the classification results by successfully classifying 96.77% of the dendritic cells. **Conclusions:** The fuzzy classification-based tools provide cell maturity and inhibition rates which help biologists evaluate severe health impacts caused by food contaminants.

## 1. Introduction

Food quality and safety are among the most important worldwide concerns, especially in France [1,2]. Mycotoxins are food contaminants that are caused by various species of molds (or fungi) [2,3,4], posing a serious public health problem [1,2,5,6,7,8,9,10,11]. Mycotoxins are found in a wide range of products which are intended for human consumption, and which can cause intoxications, chronic or acute. Eventually, even at low concentrations, these contaminants are harmful to a human’s health [6,12,13]. In this context, we can specify that many toxic effects are generated by these mycotoxins from mutagenic, teratogenic, hepatotoxic, neurotoxic, nephrotoxic, estrogenic, carcinogenic to immunosuppressive [1,2,3,4,6,8,10,11,12,13,14,15,16]. Noting that these intoxications are transmitted by skin contact, ingestion, inhalation, and breastfeeding [6]. Concerning human food intoxications due to mycotoxins, they are generally accidental, which would incite us to consider the eventual risks that could cause their presence. In addition to the demonstrated effects of mycotoxins on humans, some important aspects of toxicology have always been unknown and unexplored [7,9]. Indeed, the toxicological impact of some mycotoxins is still poorly observed or discussed [1]. In addition, the influence of mycotoxins on the human mortality rate is presumably underestimated [6]. Only with further research into understanding the effects and modes of action of mycotoxins in various species, regulations and control strategies have been put in place. Therefore, thorough and more detailed investigations by toxicologists are needed to protect humans from potential health effects induced by the presence of mycotoxins in the human food chain. This leads to refining the characterization of any risk susceptible to be generated by food contaminations by determining the doses with effect and without toxic effects, the mechanisms involved, and the target organs.

**Figure 1:**
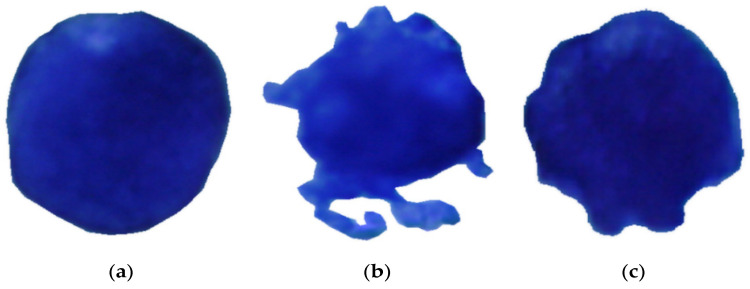
Illustration of morphological modifications during DC maturation. Immature DC (**a**) has a round shape without cytoplasmic expansions, while its more complicated and irregular shape distinguishes mature DC (**b**) with longer and finer dendrites. The morphology of dendrites changes in the presence of a toxic substance. The inhibited cell (**c**) is different from mature cells. It presents fewer and shorter extensions that appear closed with each other and smaller in size than those seen on mature cells.

Recent studies in vitro have revealed that many mycotoxins possess immunosuppressive effects, which are currently considered their most important effects [1,2,14,15,16]. This is expressed, among other things, by their capacity to alter immune reactions and, consequently, to decrease resistance to infections. Thus, the immunocompetent cells that protect the body from external agents [17] are the main targets of these toxins. Among these cells, dendritic cells (DCs), initiators of the primary immune response, play a key role in the immune system. Because of the key role of DCs, a decrease in the number of these cells or a perturbation in their maturation process due to a toxic effect can cause immunodepression. The maturation of DCs is expressed morphologically by the appearance of cytoplasmic extensions, also called dendrites, in Figure 1. No primary immune response will occur if a DC loses its ability to present antigens to T lymphocytes (LTs) in the first step of the immune reaction. Consequently, improving in vitro testing on human DCs becomes essential to detect and evaluate the adverse effects of mycotoxins on the immune system. In this context, many researchers have studied the toxic effects on the functional properties of DCs generated in vitro.

They revealed that DCs can potentially be a target, which inhibits the primary immune response [12,13,18,19,20,21,22]. Thus, they have shown that certain toxins disrupt the maturation process of immature DCs into mature DCs. Therefore, toxicological tests using immune cells involved in immune reactions and, in particular, DCs could help understand the potentially toxic effects of different food contaminants on human health. Moreover, the results of these studies allow us to determine the tolerated doses of microorganisms without causing harmful effects.

In typical cases, expert biologists adopt an assessment approach that focuses on the visual analysis of microscopic DC images. Indeed, they try to detect significant cell morphological changes between toxin-exposed and the control DC cultures. To reliably assess the undesirable effects of mycotoxins on DCs, the expert biologist must detect these cells’ most subtle morphological changes to accurately discern the different states of DCs (immature, mature, and inhibited) [12,23]. Therefore, this assessment relies on the ability of the human eye to distinguish significant morphological differences in some cases. However, this visual observation under the microscope that essentially determines their analysis for further evaluation raises many difficulties. Indeed, it requires a lot of time and careful attention from the expert biologist to detect cytoplasmic extensions and evaluate essential variations of the morphological aspects of the DCs. Thus, this work is both complex and monotonous and is susceptible to possible errors in the expert biologist’s analysis of the image. Moreover, this task is performed subjectively and is thus susceptible to inter- and intra-expert biologist variations. In other words, several experts can give different interpretation results of the same microscopic slides with an error of 10% to 15%.

Nowadays, computer vision systems’ design for automating the cell culture analysis process requires special attention. An automated analysis would allow expert biologists to facilitate the evaluation of food contaminants on human health. Indeed, these automated systems would overcome several limitations of the visual image analysis methods used until now. In particular, they would ensure speed, reproducibility, objectivity, and non-fatigability. In this context, the characterization of potential dangers caused by the presence of mycotoxins in food chains by using an automatic analysis method of microscopic images of DCs could be a reliable and efficient evaluation tool. This involves automatically counting and characterizing DCs in control and exposed cultures to collect quantitative data and relevant information that can be used for assessment. The desired automatic analysis consists of identifying, on the microscopic images of DCs, the different types of DCs (immature, mature, and inhibited) to determine if there are significant differences between the morphological characteristics corresponding to the different classes. It, therefore, allows for an observation of changes in cell morphology between these classes. This description is necessary because it will enable biologists to make a relevant assessment.

The problem of automatic cell classification from segmented microscopic images is becoming more and more common in medical image fields because the recognition of these cells in the visual examination of bone marrow and blood smears is an essential tool for identification, diagnosis, treatment, and prevention of various diseases. In our study, the classification procedure aims to discriminate the state of the dendritic cells DCs (immature, mature, and inhibited). Once the cells’ relevant characteristics (attributes) are extracted, they could be integrated into a classification process categorizing them. Indeed, based on the extracted features, the classifier categorizes or recognizes different image components. The efficient procedure of segmentation and feature extraction could greatly simplify the classifier design.

It should be noted that automatic white blood cell classification is a very complicated process compared to the human observer’s capabilities concerning regular, variable, and complex cell shapes. Given the importance of this field, various recent research has focused on providing insights into this issue [24,25]. Several relevant automatic classification techniques have been recently proposed in the literature, which can discriminate different shapes of blood cells to overcome laborious techniques and reduce workload in medical imaging analysis and diagnosis. Among these approaches, we can distinguish two types of methods depending on the availability of prior information relating to the cells to be recognized: supervised and unsupervised classification approaches.

For supervised classification, it is imperative to have a database of already labeled samples (examples of cells of each class), called *a training database*. This thus ensures the learning of the classifier. Then, a new database called *the test database* can be processed once the classifier parameters are determined. Among these algorithms, we can cite, for example, naïve Bayesian classifier (NB), Support Vector Machine (SVM), *k*-Nearest Neighbors (*k*-NNs), and Multilayer Perceptron (MLP).

The NB classifier is one of the simplest supervised learning algorithms. This classifier, which belongs to a family of simple probabilistic classifiers, allows us to categorize data by applying Bayes’ theorem with strong independence between the features. Indeed, this classifier assumes that a characteristic value is independent of any other characteristic value [26]. In the learning step, the prior probabilities of each class are calculated and then used to estimate the class of the new samples by the maximum *a posteriori* rule. The NB classifier is widely used to effectively recognize cells in microscopic blood images [26,27,28,29,30,31,32]. Despite its simplicity, Bayesian classification can often provide accurate classifications if the features are discriminative.

The *k*-NNs approach proposed by [33,34] is a regression and classification method widely used for classifying objects. This approach uses a non-parametric learning algorithm. The classification of an object by the *k*-NNs algorithm is achieved by a majority vote of its neighbors. Based on this voting, the object will be assigned to its relevant class that represents the most frequent class among its *k* nearest neighbors (*k* is a positive integer) using an appropriate metric (i.e., Euclidean distance). For cell classification in microscopic images, the *k*-NNs classifier is used to achieve good classification results [35,36,37,38,39,40,41]. In [42], authors have used the *k*-NNs classifier to discriminate between normal and abnormal (malignant) cells for acute lymphoblastic leukemia identification.

SVM is a supervised learning approach originally introduced to handle binary classification problems [43,44]. It is based on the assumption of space transformation of the input data which are not linearly separable. Indeed, SVM consists in projecting these data into a new space of higher dimension, called *feature space*, in such a way that the data become linearly separable. In this new space, the principal idea of the SVM algorithm is, therefore, to separate the data into classes by means of a margin, so that the distance between the different data groups and the margin separating them is maximum. Therefore, the SVMs goal is to determine the best separation margin (optimal hyperplane) that correctly separates the classes by as large a margin as possible. The latter designates the distance between the separation hyperplane and the closest data, called *support vectors*, delimiting the width of the margin. In general, the projection of the data into the *feature space* is performed using a kernel function. Among the most commonly used kernels for SVM, we can mention the Gaussian, polynomial, linear, and sigmoidal kernels. Concretely, the user tests the method with various kernel functions in order to choose the one that best suits his application. To solve multi-class classification problems such as the recognition of different types of blood cells, several binary classifiers have to be combined. In this context, two approaches can be distinguished: *one-against-all* and *one-against-one* (both are commonly used) [45]. The approach *one-against-all* is the simplest one to apply. It consists in training a binary SVM to discriminate the data of a class from those of the other classes. Consequently, this approach requires the construction of N binary classifiers. Recently, SVM approaches have rapidly gained more attention in cell recognition in microscopic images as they have outperformed other state-of-the-art methods in pattern recognition [30,39,41,46,47,48,49,50,51,52,53,54].

Artificial neural networks (ANNs) are undeniably one of the most recognized supervised approaches for their reliability and performance. ANNs are simple processing networks that imitate the behavior of the human brain to perform much more complex tasks. They are composed of interconnected formal artificial neurons [55]. The way these neurons are assembled together determines the architecture of an ANN model. Therefore, ANNs are distinguished by their architecture (number of inputs/outputs, number of neurons, etc.). Thus, many network architectures and learning algorithms have been proposed and are well presented in the literature [56]. Given their good results, multilayer neural networks (MNNs), also called *multilayer perceptrons* (MLPs), are currently the most widely used architectures. MLP is a simple feed-forward ANN. Its training is performed by the error gradient backpropagation algorithm which consists of modifying the network weight by minimizing a quadratic error [55]. In recent years, ANNs have been considered one of the most powerful classifiers used for pattern recognition in biomedical applications. Indeed, the most popular MLP learning algorithm has been used in many blood cells classification tasks [28,30,39,52,57,58]. In [57], authors have presented an interesting classification approach based on the combination of Fisher’s linear discriminant (FLD), also called Linear Discriminant Analysis (LDA), preprocessing method, and MLP classifier (FLD-MLP) to recognize cells in fluorescence microscopy images. To demonstrate the effectiveness of their method, the authors have compared the results obtained by their method to those obtained by the classical method of principal component analysis (PCA). Therefore, their proposed cell recognition method that used FLD as a feature selection technique was demonstrated to be more effective than the PCA technique in view of the fact that FLD maximizes the ratio of between-class to within-class scatter matrices. Based on the work published by [57], Mouelhi et al. [58] have used the FLD-MLP classifier to recognize positive- and negative-stained cell nuclei according to their textural and morphological features for automatic breast cancer detection.

Fuzzy neural network (FNN) and, more specifically, the Adaptive Neuro-fuzzy Inference System (ANFIS) is another widely used classifier for biomedical image classification. It is a very powerful method that combines ANN and fuzzy logic. Although its algorithmic complexity is higher than that of other classifiers, ANFIS contributes to better classification performance used for the early diagnosis of several diseases [59]. In [60], ANFIS was used to classify immature blood cells in leukemia patients from blood microscopic images, where it gives satisfactory results.

In contrast to supervised classification approaches, unsupervised classification methods do not require a database of labeled examples. Thus, these approaches learn by themselves and alternatively, class characterization is done from the available dataset. Among these algorithms, we can cite the K-means algorithm, the fuzzy C-means algorithm (FCM), the Expectation-Maximization (EM) algorithm, and the Mean-Shift algorithm (MS). K-means and FCM algorithms use the criterion of maximizing inter-class distances and minimizing intra-class distances to search for classes of similar objects.

We conclude the state-of-the-art classification methods used in the field of microscopic cell image analysis by giving in Table 1 the main advantages and disadvantages of these methods.

In this work, we develop classification techniques that accurately recognize DCs in microscopic images. Our approaches involve three main steps: (1) Shape characterization, (2) Dimensionality reduction, and (3) DCs classification.

The main contribution of this work is to have proposed an automatic classification of dendritic cell images. According to our knowledge, this subject has not been treated before in the literature. We first propose the SVM classification, which is classically used in studies of microscopic images of cells. Then, we propose other methods using fuzzy logic. We demonstrate that fuzzy logic is best suited for this problem. We show also that the features of the different classes of DCs often overlap and are difficult to distinguish. Consequently, the choice of descriptors is very crucial for classification, even when using automatic feature selection methods. Finally, we proposed to use fuzzy logic to propose an interpretation tool to let the biologist or the physician say the last word in the classification of doubtful cells.

The remaining sections of the paper are structured as follows: Section 2 introduces our proposed classification approaches. In Section 3, we show experimental results, and we discuss the obtained results on a collected dataset from microscopic DC images. Finally, we present conclusions and future perspectives in Section 4.

## 2. Material and Methods

In this work, we utilize the dataset described in previous work [61]. It consists of 421 microscopic DC images containing a total of 515 DCs with different cell types of which 248 are immature, 70 are mature, and 197 are inhibited. It is obvious that this database is unbalanced, particularly the mature class is considerably infrequent compared to the other two classes (minority class). To overcome the problem of unbalanced classes, we applied the classical data augmentation methods. In the proposed study, we use horizontal and vertical flipping techniques, which aim to increase the number of instances of the mature class by generating new images based on geometric transformation. Finally, the new database consists of 631 cells of which 248 are immature, 186 are mature, and 197 are inhibited.

### 2.1. Description of the Proposed Method

**Figure 2:**
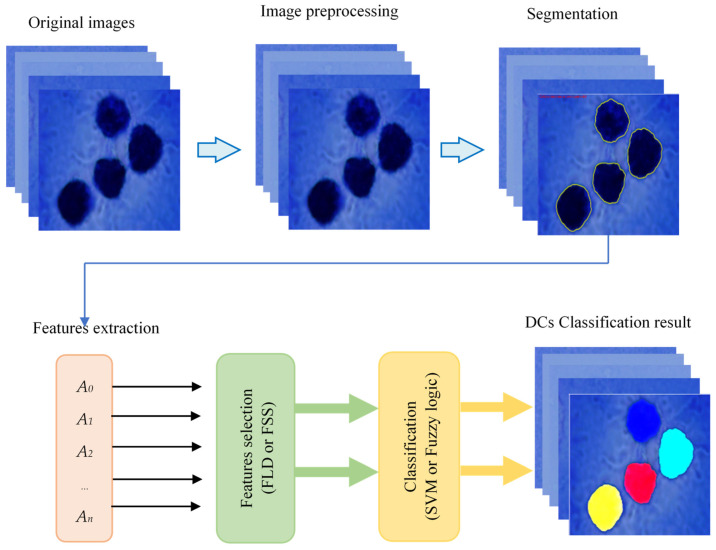
Flow chart of the proposed method of DCs classification.

Figure 2 depicts the proposed approach’s flow chart. It shows that the proposed method is primarily composed of three steps: image preprocessing and DCs segmentation, features extraction and selection, and DCs classification. To extract all DCs, cell segmentation using K-means clustering and the Chan-Vese active contour model is performed as a first step. The applied approach is detailed in [61]. In the second step, geometric characteristics used to distinguish between different types of DCs are extracted based on the shape of the cell contour obtained during the first step. After that, a feature-selection technique based on FLD is used to determine the relevant attributes. Finally, two classifiers (SVM and Fuzzy Logic) are used to classify DCs into their appropriate categories (mature, immature, and inhibited).

### 2.2. Shape Characterization

The shape characterization step aims to obtain descriptors or attributes describing the shape of segmented cells. These attributes will be used as input for the classification process. Formally, shape characterization can be defined as a function f which maps the segmented cell SC to a features vector x, that is,$\;f:SC\to x=\left(x_{1},x_{2},\ldots ,x_{d}\right)$ where d is the number of attributes used to characterize the segmented cell [62]. The most recent works of literature proposed to classify cells in microscopic images have focused on texture, size, and shape features. Generally, the features used in white blood analysis can be grouped into textural and geometric features [63,64]. To classify DCs in microscopic images, expert biologists rely mainly on geometric characteristics (Table 2) to distinguish between their different types (mature, immature, or inhibited). In this application, the texture information is not important and only the shape of the contour is used for characterization. Therefore, we propose to use the contour deformation analysis to define the class to which a cell belongs. Indeed, immature DCs, that is to say, having not yet encountered antigens, are approximately round and do not possess dendrites. Only small protuberances can appear on the surface of these cells after a few days of culture. On the other hand, in the mature state, they have captured an antigen; DCs are characterized by their more complex irregular shape and relatively fine and long dendrites. However, in the presence of a more or less toxic substance, the morphology of the cytoplasmic expansions is different. The more or less inhibited cells have a different appearance than the mature control cells. They have fewer and shorter extensions. In other words, these extensions seem clogged together, and their size is smaller than the sizes observed on the mature control cells.

Therefore, depending on the deformation of the cell contour, it could be assigned to one of the three following classes:

- Class IM: immature DC.
- Class MA: mature DC.
- Class IN: inhibited DC.

Table 3 presents an example of the morphological attributes extracted from some segmented cells as well as the class assigned by an expert biologist.

### 2.3. Dimensionality Reduction

The dimensionality reduction step aims to reduce the dimensionality of the vectors associated with the segmented cells by determining the most relevant, discriminating, and uncorrelated attributes. This step is a commonly used process in machine learning and pattern recognition. Dimensionality reduction techniques can be categorized into filter methods, wrapper methods, and embedded methods [62,65]. Indeed, it is essential to filter the feature vectors by eliminating redundant and irrelevant features to improve classifier performance. We propose to use the automatic dimensionality reduction method for better class discrimination. To provide better and more representative descriptors of the DCs’ shapes, we have chosen to apply the FLD method to the input data before entering the classification phase. This method looks for the vectors in the underlying space that best distinguish among the different classes (rather than best describe the data). More formally, given a number of independent features describing the data, FLD generates a linear combination of these, which yields the largest mean differences between the wanted classes. Mathematically, we define two measures for all the samples of all categories. These two measures, called *within-class* scatter matrix and *between-class* scatter matrix, are given by Equations (1) and (2), respectively:

(1) $$S_{w}=\overset{c}{\underset{j=1}{\sum }}\overset{N_{j}}{\underset{i=1}{\sum }}\left(x_{i}^{j}-\mu_{j}\right){\left(x_{i}^{j}-\mu_{j}\right)}^{T}$$

where $x_{i}^{j}$ is the $i^{th}$ sample of class j, $\mu_{j}$ is the mean of class j, c is the number of classes, and $N_{j}$ is the number of samples in class j.

(2) $$S_{b}=\overset{c}{\underset{j=1}{\sum }}\left(\mu_{j}-\mu \right){\left(\mu_{j}-\mu \right)}^{T}$$

where μ is the mean of all classes.

FLD aims to minimize the *within-class* (intra-class) measure while maximizing the *between*-class (inter-class) measure by maximizing the following ratio:

(3) $$J\left(w\right)=\frac{w^{T}S_{b}w}{w^{T}S_{w}w}$$

The linear transformation w is obtained by the eigenvectors of the matrix $S_{w}^{-1}S_{b}$. The dimension of the training data set is reduced to two variables in our case (F1 and F2). The new attributes of the training database $F_{in}={\left(F1,F2\right)}^{T}$ are calculated using the following transformation of the original data $X_{in}$:

(4) $$F_{in}=X_{in}w$$

The originally considered attributes (Table 2) may be correlated, and the cells of the different classes in the database sometimes present a substantial similarity. We propose to use FLD to obtain new independent variables representing the cells and maximize the difference between the classes in the learning database. The FLD preprocessing method is also used to reduce the input dataset’s dimension and generate independent components that are generally more appropriate for classification purposes than the original features. Indeed, dimensionality reduction, which eliminates irrelevant attributes, allows us to obtain similar or even better recognition rates [58].

### 2.4. DC Classification Using SVM Classifier

**Figure 3:**
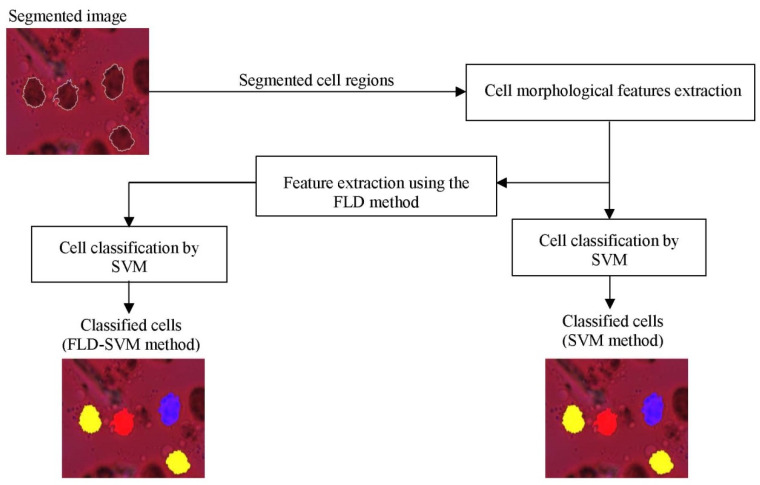
Examples of the classification results of dendritic cells by the SVM classifier with (FLD-SVM) and without (SVM) dimensionality reduction. The classified cells are marked by the following color masks: immature cell (yellow), mature cell (red), and inhibited cell (blue).

For DC classification between the different classes (mature, immature, and inhibited), we propose first to use the support vector machine (SVM) classifier in two different ways: with and without dimensionality reduction. SVM has been an efficient and powerful tool for solving many classification problems, particularly cell recognition in microscopic images [30,39,41,51,52,54,66,67]. Given a microscopic image of DC, the segmentation method described in our previous work [61] is used to have cell contour. Features calculated using the FLD method described previously are then presented to the SVM classifier to discriminate between the three classes. Figure 3 represents the SVM and FLD-SVM classifiers diagrams with a DC image example.

### 2.5. DC Classification Using Fuzzy Logic

Fuzzy logic could be defined as the continuity of the Boolean logic that Lotfi Zadeh developed in 1965 [68]. The latter relied on his mathematical theory of fuzzy sets for its creation, considered an extension of classical set theory. By introducing the concept of degree in verifying a condition that could be in a state other than true or false, fuzzy logic gives appreciable flexibility to the reasonings that adopt it. In this work, we are interested in its use in the framework of classification. Thus, fuzzy set theory [68] could allow us to define the gradual membership of each segmented cell to a given class C. This membership is defined by a membership function $\mu_{C}$ whose values are in the interval [0, 1]. For a given cell, the classifier results are represented by the fuzzy set $\left\{\mu_{IM},\;\mu_{MA},\;\mu_{IN}\right\}$ where $\mu_{IM}$ denotes the degree of membership in the immature class, $\mu_{MA}$ in the mature class and $\mu_{IN}$ in the inhibited class.

#### 2.5.1. Fuzzy Inference System (FIS)

**Figure 4:**
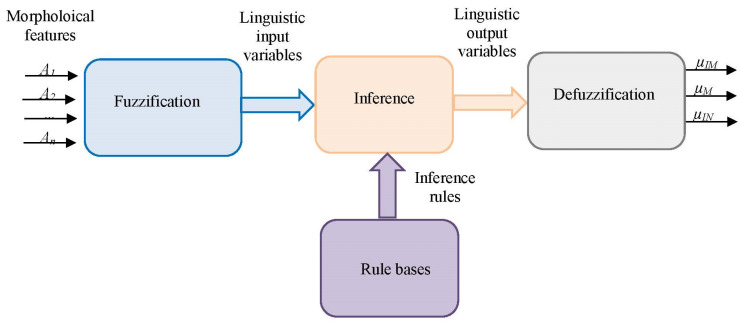
Block diagram of the fuzzy classification system.

A fuzzy classification system consists of three main stages [69] illustrated in Figure 4: fuzzification, fuzzy inference, and defuzzification, which we will further describe below.

◾ Fuzzification

The first step of fuzzification is to determine the degree of membership of a morphological feature value to a fuzzy set. Indeed, this value provides a way to convert the real domain into a fuzzy domain. The degrees of membership values range from 0 to 1. The membership functions are among the most important elements to find in the fuzzification process. In fact, many types of membership functions can be found in the literature, with the Gaussian, the trapezoidal, and the triangular functions being the most common and most used membership functions [69].

◾ Fuzzy inference

Fuzzy inference, also called inference engine, is the fundamental block of the fuzzy system. The inference engine emulates the behavior of the human expert by using his knowledge to interpret the classifier better and thus control it well. Once the rules to be applied have been decided, this step consists of defining the degrees of membership of the output variable to the fuzzy sets. The two most commonly used fuzzy inference systems in the literature are Mamdani [70] and Sugeno [71].

(1) The Mamdani method (*max* min inference method)

The Mamdani fuzzy model is composed of fuzzy rule bases of the following form [72]:

(5) $$If\;x_{1}\;is\;A_{1}\;and\;x_{2}\;is\;A_{2}\;and\ldots \;x_{n}\;is\;A_{n}\;\;\;\;\;Then\;\;\;\;\;y_{1}\;is\;B_{1}\;and\;y_{2}\;is\;B_{2}\;and\ldots \;y_{m}\;is\;B_{m}\;Premise\;\left(or\;antecedent\right)\;\;\;\;\;\;\;\;\;\;\;\;\;\;\;\;\;\;\;\;\;\;\;\;\;\;\;\;\;\;\;conclusion\;\left(or\;consequent\right)$$

with $x_{n}$ et $y_{m}$ being the linguistic input and output variables and $A_{n}$ et $B_{m}$ the linguistic fuzzy sets associated with them.

In the case of inference engines based on the Mamdani method, the degrees of membership of the output variable are computed through the AND and OR operators using the *min* and *max* functions.

(2) The Takagi–Sugeno method (sum-prod inference method)

Similar to the previous method, the Takagi–Sugeno fuzzy model uses conditional clauses in which the premise is imperatively a linguistic variable, but the conclusion is a numerical variable. The conclusion could be calculated using any formula or mathematical function depending on the result and desired behavior (e.g., polynomials, constants).

(6) $$If\;x_{1}\;is\;A_{1}\;and\;x_{2}\;is\;A_{2}\;and\ldots \;x_{n}\;is\;A_{n}\;\;\;\;\;Then\;\;\;\;\;y_{m}=f\left(x_{1},\;x_{2},\ldots ,x_{n}\right)$$

In the literature, $f_{m}$ are usually linear functions [72].

◾ **Defuzzification**

This is, in fact, the last step of the fuzzy system. Certainly, the outputs of the inference engine represented as degrees of membership to the functions of the output must be converted and thereafter applied on the classifier. In this case, we can talk about defuzzification which is the conversion of these fuzzy values into usable real variables. There are three basic methods of defuzzification depending on the shape of the desired output and the type of membership functions of the output:

- Center of gravity method;
- Maxima method;
- Bisector method.

#### 2.5.2. Proposed Fuzzy Systems

We propose two fuzzy DC classification approaches based on the FIS structure shown in Figure 4.

**Figure 5:**
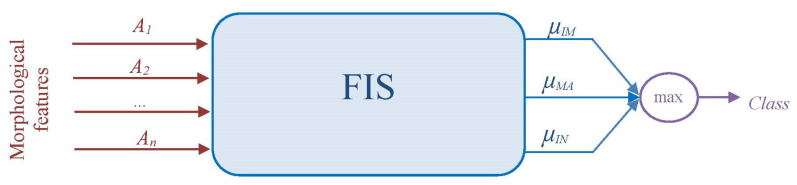
Flowchart of the proposed FIS1 system for fuzzy DCs classification.

**Figure 6:**
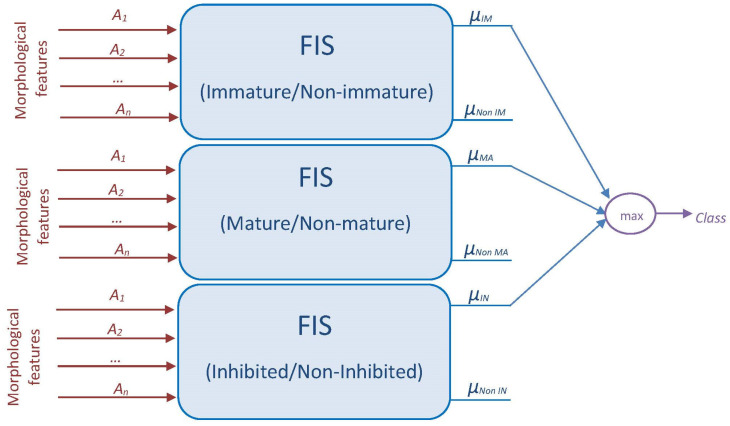
Flowchart of the proposed FIS2 system for fuzzy DCs classification.

For a first system (FIS1), we used a single FIS to identify the three classes (Figure 5). Then, we propose a second system (FIS2) in which the classification process is proposed by combining the results from three FISs (Figure 6). The last system consists of using one classifier per cell type. In other words, each binary classifier will separate each class from the other two classes (Immature/Non-immature, Mature/Non-mature, and Inhibited/Non-inhibited).

## 3. Results and Discussions

### 3.1. Classification Results Using SVM and FLD-SVM Classifiers

**Figure 7:**
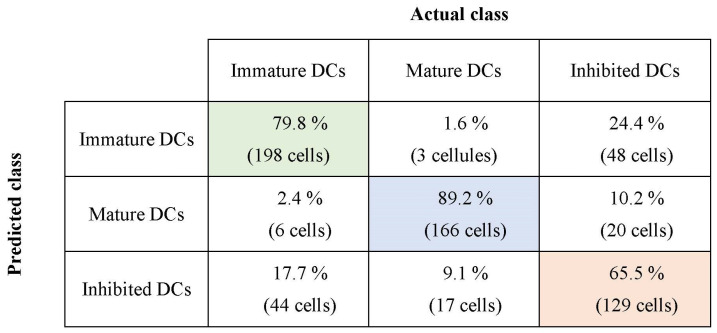
Confusion matrix for the three different classes of DCs using the FLD-SVM method.

This section presents a comparative study of the classification results using the SVM method with those obtained by the FLD-SVM classifier. FLD-SVM is a combination of the FLD feature extraction method and the SVM classifier. FLD is used to generate new uncorrelated and relevant features from the input dataset, and then, SVM is used to classify these newly obtained components. SVM is the classical support vector machine trained with the original DC features extracted from a set of training images. In our experimental study, three binary SVMs, with Gaussian kernel function are designed to estimate the cell class. To evaluate the performance of the used recognition method, we use a cross-validation technique [73]. This latter was also used to choose the Gaussian kernel giving our dataset’s best performance compared to the other kernels mentioned before. For experiments, the leave-one-out cross-validation (LOOCV) approach is applied. At each step, one cell is used for the test and the remaining examples are for training. This procedure is performed N times, where N is the number of cells in the dataset. Table 4 reports the obtained correct classification rates according to the DC class assigned and the classifier used. The SVM and FLD-SVM reach 75.75% and 78.13% of the correct classification rate, respectively. In other words, the SVM method correctly classifies (195/248) immature cells, (160/186) mature cells, and (123/197) inhibited cells, while the FLD-SVM method correctly classifies (198/248) immature cells (166/186) mature cells and (129/197) inhibited cells. To better illustrate the results obtained by the FLD-SVM classifier, the confusion matrix for the three different classes of DCs is presented in Figure 7. The presented results show that the feature extraction improves recognition accuracy. Moreover, we can clearly observe a good classification between immature and mature cells and many errors between immature and mature on one side and inhibited cells on the other side. From these results, we can see that this classifier gives perfect classification rates. The errors come mainly from inhibited cells that were classified as immature and mature cells. Indeed, 48 inhibited cells were classified as immature, and 20 cells were classified as mature. Some inhibited cells are classified as immature because the toxicant prevents them from maturing and, therefore, remains at a stage that corresponds to immaturity when they should be maturing. In addition, 44 immature cells were classified as inhibited cells. Consequently, the FLD-SVM misclassifies these DCs since their characteristics are relatively close. As a result, it is not easy to differentiate between them precisely using SVM, which provides poor recognition results. Therefore, the results we have just presented illustrate that the classical (boolean) classification of DCs with similar characteristics is difficult. This is why, in the following, we propose the use of fuzzy classification approaches to integrate class imprecision and to define confidence measures [74].

### 3.2. Classification Results Using Fuzzy Inference System (FIS)

**Figure 8:**
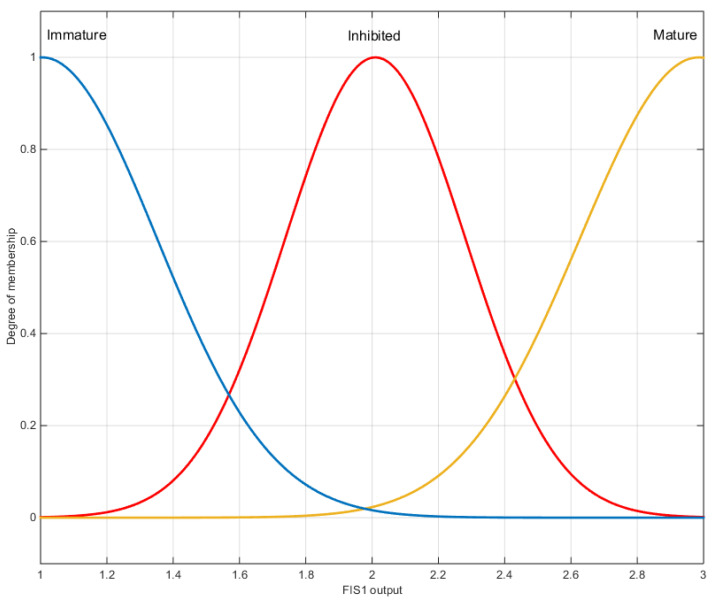
Membership functions of the fuzzy sets of the FIS1 system output.

We used Matlab’s Fuzzy Toolbox to generate the fuzzy inference systems. In the fuzzification step, we estimated the membership functions from the feature histograms. We implemented Mamdani FIS. To automatically generate the fuzzy rules of the system, we also used the FCM method. The defuzzification method used is the maxima method for the FIS1 system and the center of gravity method for the FIS2 system, with Gaussian membership functions for the output (Figure 8).

**Figure 9:**
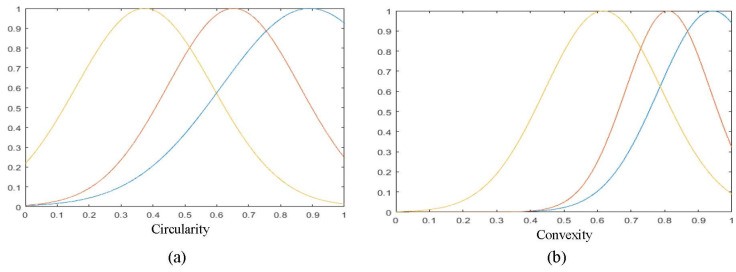
Membership functions used in the fuzzification step for the Circularity (**a**) and Convexity (**b**) inputs (blue: immature, red: inhibited, and yellow: mature).

The cross-validation method is used to find the optimal training data for the construction of the FISs. For the input features, we experimented with three scenarios. For the first scenario, we used all original features at the classifier’s input. Then, in the second one, we used the FLD technique to select the relevant features. Finally, we analyzed the membership functions of different features and found that they overlap more than some individual features. For this reason, we experiment the feature subset selection in order to improve the results. Indeed, in order to reduce the dimensionality, we can proceed in two ways. Feature extraction is the first way, it aims to provide new features from linear or non-linear transformations of the original features, and such is the case of the FLD method. The second way is to select the optimal subset of features from the original attributes. To reduce the dimensionality to two (as with FLD), we chose the two original features corresponding to the maximum eigenvalues of the Fisher matrix $S_{w}^{-1}S_{b}$ (Equations (1) and (2)) which aims to minimize the *within-class* measure while maximizing the *between-class* measure. This resulted in the selection of two features, circularity and convexity. We show the corresponding membership functions in Figure 9. We note this method by FSS (Feature Subset Selection).

First, we will consider the FIS1 system which is configured to classify cells into the three classes directly. Using all the features described in Section 2.1, the FIS1 system succeeds in classifying 62.03% of the cells. Using the features selected by the FLD algorithm (Section 2.2), only 61.73% of cells are correctly classified. In both cases, the class of inhibited cells poses a problem as with the SVM classification. Finally, the classification based on FSS features led to 65.27% correct classified cells with a good improvement in the classification of inhibited cells. The correct classification rates of the three classes with the FIS1 system are reported in Table 5.

The FIS2 classification system is made up of three classifiers which separate the three classes from one another each time. Classification rates with this system were better than with the first system. With all original features given in the input, 82% of cells are classified correctly. With the FLD extraction, the correct classification rate is 78.86%. Finally, using FSS, the correct classification rate rises to 96.77%. The different rates obtained with the SIF2 system are given in Table 6.

Our proposed model (FSS + fuzzy classification) brought the best performance to the problem posed. The features of the different classes of DCs often overlap and are difficult to distinguish. For this reason, classical classification methods such as SVM did not provide reliable cell recognition. However, the classification based on fuzzy logic allows us to take into account the uncertainties and inaccuracies of insufficiently known data. Since the features had very large intersection areas, especially with the inhibited class, the fuzzy system with FSS was best suited for separation.

The first limitation of the proposed method is that it failed to classify all cells correctly. There is still confusion between inhibited and mature cells. With FIS2 + FSS, we reached better performances, but these two most difficult classes were not totally differentiated. For this reason, we propose in the next section a help tool that would present the unclear results to the biologist who will decide at the end in case of doubt. Another limitation of the proposed method is that we have chosen the shape characteristics ourselves. We then showed that some features had no contribution; on the contrary, some of them deceived the system. It would be interesting to exploit deep learning methods that extract relevant features automatically. It is regretful that we could not experiment the methods on our base which is composed of a very limited number of images. In future, we could study the combination of deep learning classification and fuzzy logic for parameter control as done in [75,76].

### 3.3. Interpretation Aiding Tools

In our study, the features of the different classes of DCs often overlap and are difficult to distinguish. For this reason, classical classification methods such as SVM do not provide reliable cell recognition (79.8% of immature cells, 89.2% of mature cells, and 65.5% of inhibited cells). Therefore, we have proposed the classification based on fuzzy logic which allows us to take into account the uncertainties and inaccuracies of insufficiently known data. Fuzzy classification can also be used to propose a system to help in the evaluation of the harmful effects of food contaminants with confidence indices. In other words, we will take from the outputs of the inference systems the values of the membership functions of each class and let the biologist make the final decision on the class of the cell. The values of membership to the three classes can be interpreted as a degree of immaturity, degree of maturation and degree of inhibition. The degree of inhibition will be used, for example, by the biologist to assess the effects of toxic products on the immune system.

**Figure 10:**
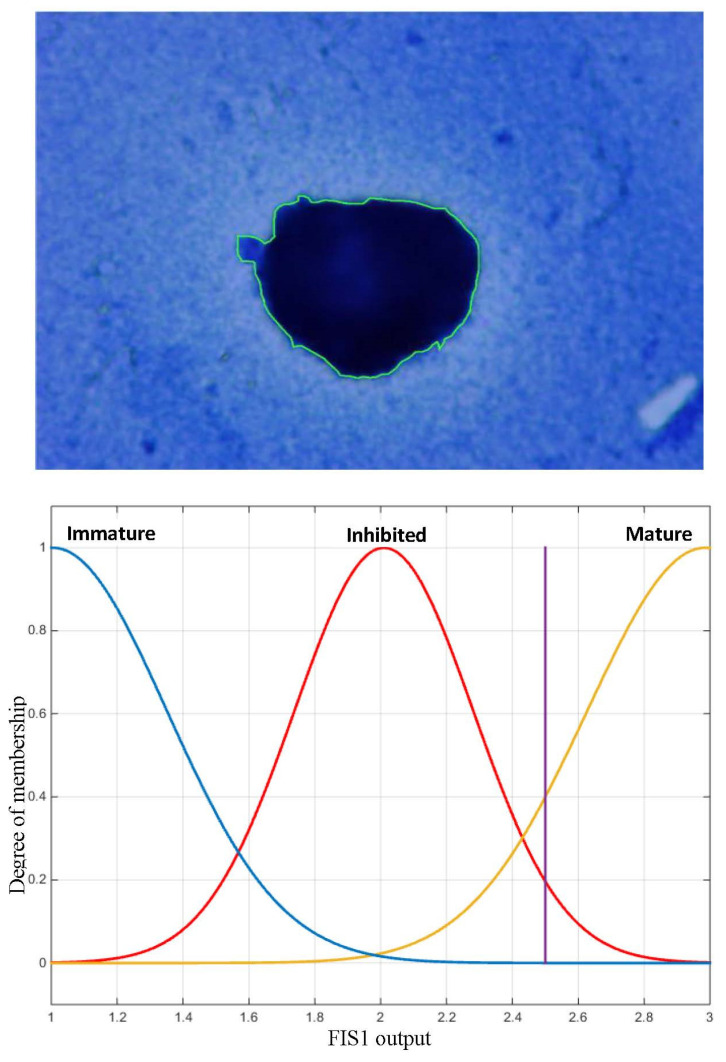
Membership functions of fuzzy sets of the FIS1 output. The vertical line corresponds to the defuzzification of the input corresponding to the cell. According to this curve, this cell is 20% inhibited and 40% mature.

We illustrate in Figure 10 an example of the output of the FIS1 system for a cell which raises doubts about the classification. The 2.45 output value shown by the vertical bar with the output membership functions indicates that this cell is 20% inhibited and 40% mature. These rates will help the biologist to determine the type of cell and assess the effect of the toxicant.

**Figure 11:**
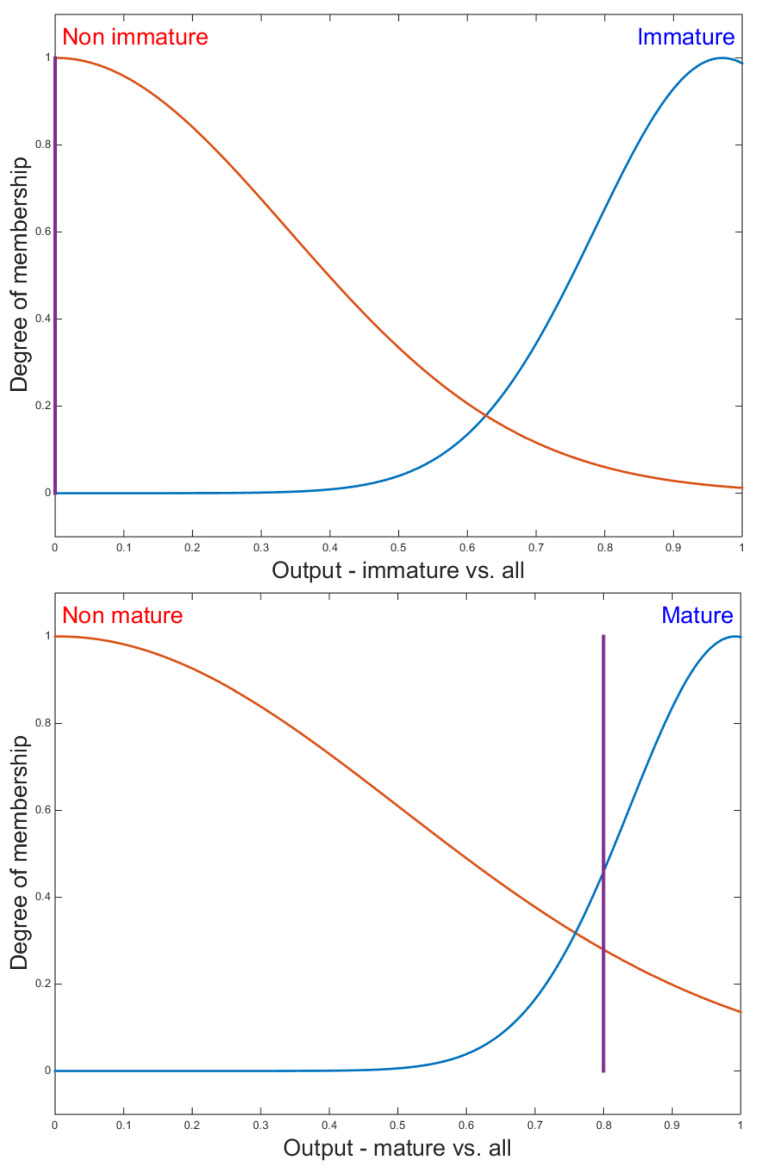

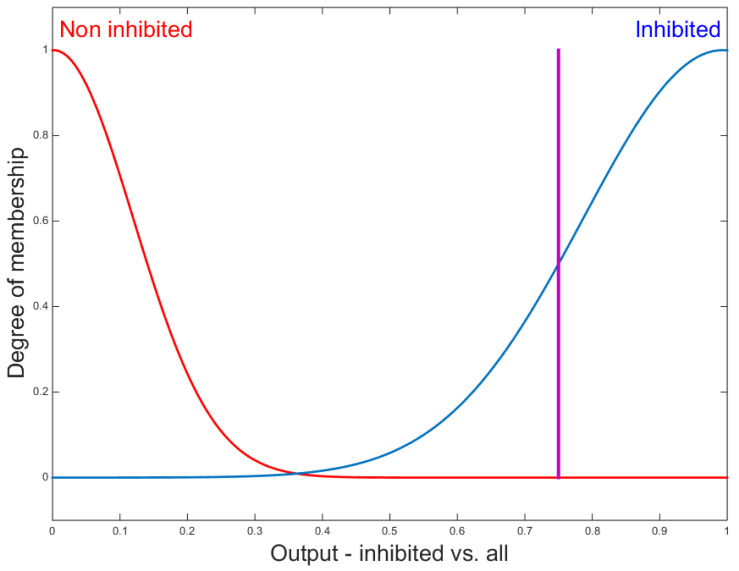
Membership functions of fuzzy sets of the FIS2 outputs. The vertical line corresponds to the defuzzification of the input corresponding to a 50% inhibited and 46% mature cell.

The outputs of the three FIS2 inference systems for the same input cell are shown in Figure 11. In this figure we see that this cell is 0% immature, 46% mature, and 50% inhibited.

## 4. Conclusions and Perspectives

This paper has designed and experimented with classification techniques to recognize dendritic cells based on extracted and selected morphological cell features. Classification is addressed to identify immature, mature, and inhibited cells for a posteriori evaluation of the toxic effects of some contaminants on the immune system. The first proposed classifier is based on SVM and FLD to select uncorrelated and relevant attributes. The FLD-SVM classifier does not give satisfactory results due to the significant confusion between the inhibited cells on one side and the other two cell types (immature and mature). Subsequently, a second strategy was developed to improve dendritic cell recognition results in microscopic images. This one is based on fuzzy logic, which allows us to consider the uncertainties and imprecision of the data. The classification results were much improved with the fuzzy classification system (FIS2 + FSS), which successfully classified 96.77% of the cells. Classification results could be improved by combining fuzzy logic with a deep learning classifier, but to do so, we will need a larger image base. The constructed fuzzy classification system has also been proposed to provide biologists with decision support tools for dendritic cell recognition in uncertainty-type cell identification. These fuzzy classification-based tools also provide cell maturity and inhibition rates to help biologists evaluate the severe health impacts of food contaminants.

Finally, we note that this work is proposed in the context of processing and analyzing 2D microscopic images of dendritic cells. However, 3D image analysis could be considered to provide biologists with additional clues about the toxic effects of food contaminants. The analysis of the three-dimensional aspect of dendritic cells that starts with the 3D acquisition of these cells is the perspective of the work presented in this paper.

## Figures and Tables

**Table 1:** Microscopic cell image classification methods overview: advantages and limitations.

Classifier	Advantages	Limitations
NB classifier [26,27,28,29,30,31,32]	- Fast and efficient - Simple and easy to implement - Uncorrelated features can be reliably recognized.	- Presumes that all features are independent, which is rarely the case
SVM [30,39,41,46,47,48,49,50,51,52,53,54]	- High accuracy for cells classification - Effective for non-linearly separable datasets	- It remains difficult to identify the appropriate kernel function - The hyperplane choices and kernel parameters influence accuracy and performance - Large datasets require a long training time
*k*-NNs classifier [35,36,37,38,39,40,41,42]	- Simple and easy to implement - Recognizes cells with high accuracy	- It is difficult to determine the optimal value of *k* - Sensitive to irrelevant features
MLPs [28,30,39,52,57,58]	- Performs highly accurate recognition of cells - Used to resolve difficult nonlinear issues	- It is a matter to determine the number of neurons and layers. - Requires extensive computational time
ANFIS [59,60]	- Powerful classifier that combines advantages of ANN and fuzzy logic - Requires fewer adjustable parameters	- It is challenging to identify appropriate membership functions - Require high computational complexity

**Table 2:** Morphological features used to characterize dendritic cell shape.

No.	Feature	Symbol/Formula
**1**	Area	$Area=\underset{x\in SC}{\sum }1$
**2**	Perimeter	$\mathrm{Perimeter}=\underset{x\in B}{\sum }1$
**3**	Circularity	$\mathrm{Circularity}=\frac{4\pi Area}{{\mathrm{Perimeter}}^{2}}$
**4**	Convexity	$\mathrm{Convexity}=\frac{Area}{ConvexArea}$
**5**	Eccentricity	$\mathrm{Eccentricity}=\frac{\sqrt{L^{2}-l^{2}}}{L}$
**6**	Elongation	$\mathrm{Elongation}=\frac{l}{L}$

**Table 3:** Numerical values of the morphological characteristics of sample cells segmented and classified by the expert biologist.

Attribute	Cell 1 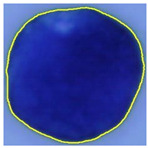	Cell 2 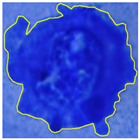	Cell 3 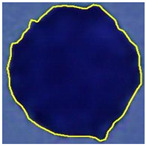
Area	58,888	99,755	38,897
Perimeter	870.4980	1556.5	732.0330
Circularity	0.9766	0.5174	0.9121
Convexity	0.9924	0.8797	0.9757
Eccentricity	0.3875	0.4294	0.5271
Elongation	1.0847	1.1073	1.1768
**Class**	**Immature**	**Mature**	**Inhibited**

**Table 4:** Different classification rates obtained by the SVM and FLD-SVM classifiers on the considered experimental dataset.

Classifier	Immature DCs	Mature DCs	Inhibited DCs
SVM	78.6%	86%	62.4%
FLD-SVM	79.8%	89.2%	65.5%

**Table 5:** Correct classification rate of the three classes with the FIS1 system.

	Immature DCs	Mature DCs	Inhibited DCs
FIS1 (all features at the input)	91.1%	67.1%	27.9%
FIS1 + FLD	83.3%	76.3%	25.6%
FIS1 + FSS	86.7%	68.4%	40.7%

**Table 6:** Correct classification rate of the three classes with the FIS2 system.

	Immature DCs	Mature DCs	Inhibited DCs
FIS2 (all features at the input)	96.7%	73.7%	75.6%
FIS2 + FLD	91.1%	71.1%	74.4%
FIS2 + FSS	100%	96.1%	94.2%

## Data Availability

The data presented in this study are openly available in Zenodo repository at https://doi.org/10.5281/zenodo7501891 (access on 20 December 2022).
